# High-flow tracheal therapy vs. tracheostomy mask in weaning: insights from a propensity-matched cohort

**DOI:** 10.1186/s12890-025-04037-6

**Published:** 2025-11-21

**Authors:** Ana G. López-Rubio, Yennifer Torres-Suárez, Diana C. Ortiz-Moreno, Camilo A. Pérez-Velázquez, Laura M. Castillo-Morales, Andrés Felipe Mora-Salamanca, Jorge I. Alvarado-Sánchez

**Affiliations:** 1 Department of Critical Medicine and Intensive Care, Fundación Santa Fe de Bogotá, Bogotá, Colombia; 2 Faculty of Medicine, Universidad del Rosario, Bogotá, Colombia; 3 Department of Physiology, Faculty of Medicine, Universidad Nacional de Colombia, Bogotá, Colombia

**Keywords:** Prolonged weaning, Tracheostomy, High-flow tracheal therapy, Mechanical ventilation, Weaning duration

## Abstract

**Background:**

Difficult weaning from mechanical ventilation remains a major challenge in intensive care units (ICUs), particularly among patients requiring prolonged respiratory support. High-flow tracheal therapy (HFTT) may improve gas exchange, humidification, and comfort in tracheostomized patients, but its clinical impact on weaning duration is uncertain.

**Methods:**

We conducted a retrospective cohort study including critically ill adult patients who underwent tracheostomy between 2020 and 2023. Patients received either HFTT or conventional oxygen therapy via tracheostomy mask. The primary outcome was post-tracheostomy mechanical ventilation duration, defined as the number of days from tracheostomy to successful ventilatory support withdrawal. Propensity score matching was performed using age, sex, Sequential Organ Failure Assessment (SOFA) score, presence of acute respiratory distress syndrome (ARDS), and sedation duration. Between-group comparisons employed parametric or nonparametric tests as appropriate, and a negative binomial regression model assessed potential associations with weaning duration.

**Results:**

Of 207 screened patients, 65 were included after matching (HFTT group = 19, tracheostomy mask group = 46). Baseline characteristics were balanced. The median post-tracheostomy weaning duration was 13 [8–19] days in the HFTT group and 11 [6–17] days in the tracheostomy mask group (*p* = 0.47). Total mechanical ventilation time was longer in the HFTT group (*p* = 0.02), but HFTT use was not associated with the post-tracheostomy weaning duration (β = 0.01 ± 0.22, *p* = 0.96).

**Conclusions:**

In this matched cohort, high-flow tracheal therapy did not shorten the weaning duration after tracheostomy compared with conventional oxygen therapy. Prospective studies are needed to clarify its optimal clinical role.

**Supplementary Information:**

The online version contains supplementary material available at 10.1186/s12890-025-04037-6.

## Background

Difficult weaning from mechanical ventilation is a frequent and clinically relevant challenge in intensive care units, especially among patients requiring prolonged respiratory support. Most of the time spent on mechanical ventilation occurs during the weaning process [1]. In critically ill patients, prolonged weaning (≥ 7 days) is associated with an increased risk of adverse outcomes such as increased mortality and higher healthcare costs [1, 2].

Tracheostomy is commonly performed in mechanically ventilated patients to increase comfort, reduce sedation requirements, and facilitate weaning [3]. However, most studies assessing the impact of tracheostomy on weaning duration have focused primarily on the timing and technique of the procedure. Few have evaluated strategies to optimize ventilatory support after tracheostomy in patients with difficult or prolonged weaning [4].

Compared with endotracheal tubes, tracheostomies alter airway mechanics by reducing upper airway resistance and dead space volume [3, 5]. Nonetheless, when compared to the native airway, the tracheostomy tube increases the resistive work of breathing due to elevated tidal volumes and transdiaphragmatic pressures [3]. Additionally, the influx of cold and dry air through the cannula can worsen airway physiology. These factors may impair spontaneous breathing tolerance, particularly in patients with diaphragmatic dysfunction or prolonged mechanical ventilation.

Among the available oxygenation strategies after tracheostomy, high-flow tracheal therapy (HFTT) has been proposed as a potentially beneficial alternative. High-flow systems deliver warm, humidified air at flow rates of up to 60 L/min [6], improving oxygenation and mucociliary function, reducing dead space and airway resistance, and creating a flow-dependent increase in airway pressure [7–10].

Although these physiological effects are well documented in nasal high-flow therapy, their applicability in tracheostomized patients remains unclear. Preliminary studies suggest that HFTT could improve comfort and ventilatory efficiency in this population [11, 12]. However, its clinical impact on ventilator weaning duration has not been rigorously evaluated [6, 9].

Therefore, we conducted a retrospective cohort study of tracheostomized patients treated with either tracheostomy mask or HFTT, aiming to assess their association with the duration of mechanical ventilation after tracheostomy.

## Methods

### Study design

A retrospective cohort study was conducted. This paper was prepared following the recommendations outlined in the Strengthening the Reporting of Observational Studies in Epidemiology (STROBE) guidelines [13].

### Sample

All adult patients (≥ 18 years) admitted to the ICU between 2020 and 2023 who underwent tracheostomy during their ICU stay were eligible for inclusion.

Patients were excluded if they (1) had a preexisting tracheostomy prior to ICU admission, (2) had incomplete medical records, or (3) received both HFTT and tracheostomy mask during the weaning process.

### Variables

We collected demographic and clinical data from the institutional electronic health record system. The exposure was defined as receiving either HFTT or conventional oxygen therapy post-tracheostomy. The primary outcome was post-tracheostomy mechanical ventilation duration, defined as the number of days from tracheostomy insertion to successful mechanical ventilation withdrawal. This was assessed through a spontaneous breathing trial using the following ventilatory parameters: pressure support and a positive end-expiratory pressure ≤ 8 cm H2O. A patient was considered ready for extubation if they met the following criteria: hemodynamic stability, stable exhaled tidal volume, respiratory rate less than 30 breaths per minute, and no clinical signs of muscle fatigue or use of accessory respiratory muscles.

Other demographic and clinical variables were assessed to identify potential associations with either the exposure, the outcomes or both. Among the variables collected we included age, sex, severity scores (Sequential Organ Failure Assessment [SOFA], Acute Physiology and Chronic Health Evaluation II [APACHE-II], Simplified Acute Physiology Score II [SAPS II]), acute respiratory distress syndrome (ARDS) diagnosis according to the Berlin criteria, vasopressor requirement and length, extubation failure (the need for reintubation within 48 h of extubation), early tracheostomy (a tracheostomy performed within the first 7 days of mechanical ventilation), number of days under sedation, neuromuscular blockers use and mechanical ventilation duration.

### Procedures

Patients requiring prolonged mechanical ventilation who underwent tracheostomy were managed in accordance with institutional guidelines. The decision to initiate either HFTT or conventional oxygen therapy via tracheostomy mask was made by the attending intensive care physician and the respiratory therapy team. Although both modalities are recognized within our institutional practice, no institutional standardized protocol is applied. The choice was based on clinical judgment, patient tolerance, availability of equipment, and anticipated difficulty in weaning.

HFTT was delivered at gas flow rates between 20 and 60 L/min, heated to 37 °C, and fully humidified using the AIRVOTM 2 system (Fisher & Paykel Healthcare, Auckland, New Zealand). The therapy was administered via an interface specifically designed for tracheostomy cannulas, with the cuff inflated (Optiflow+, Fisher & Paykel Healthcare, Auckland, New Zealand). Conventional oxygen therapy was delivered through a tracheostomy mask connected to a high-volume nebulizer, with flow rates adjusted between 6 and 15 L/min. Regardless of the oxygen delivery method, these treatments were alternated with ventilator support sessions as tolerated by the patient, in line with the local weaning strategy.

Across ICUs (cardiovascular, sepsis/respiratory, neurological, surgical and burns), modality selection could be influenced by local practice patterns and logistical factors. During the study period, no service-level default protocol mandated either modality. Device availability (limited number of AIRVO™ 2 systems), staffing considerations (24/7 respiratory therapy coverage), and humidification standards (active heated humidification for HFTT vs. large-volume nebulization for mask therapy) occasionally constrained the choice of modality. Neurological and surgical ICUs more frequently adopted HFTT as part of early weaning initiatives, whereas medical ICUs—where ARDS cases were more common—predominantly used tracheostomy masks. Transition thresholds from ventilatory support to spontaneous breathing were uniform across units, consisting of daily screening for spontaneous breathing trials (pressure support and PEEP ≤ 8 cmH₂O), but patient tolerance often determined whether HFTT or a mask was used. No formal decannulation or capping protocol was in place, and additional speech-language therapy was determined on a case-by-case basis depending on secretion burden and clinical stability.

Alternative methods such as the T-piece or speaking valve were not routinely used during the study period and were not included in the analysis.

### Statistical analysis

A logistic regression model was used to estimate the propensity score, representing the probability of receiving HFTT based on baseline characteristics. The dependent variable was the use of HFTT versus conventional oxygen therapy, while the potential confounders included age, sex, SOFA score, presence of ARDS, and duration of sedation. Patients were matched using nearest-neighbor matching without replacement with a 3:1 ratio (control: treatment) and a caliper of 0.2 on the logit of the propensity score. Exact matching was applied for sex to ensure same-gender pairing. Covariate balance between groups was assessed using standardized mean differences (SMDs) and variance ratios (VRs), with SMD < 0.1 and VR ≈ 1 indicating adequate balance. Propensity score distributions were visually inspected using kernel density plots before and after matching.

To ensure the stability of the propensity score model, multicollinearity among the independent variables was examined using the variance inflation factor (VIF).

In the matched dataset, continuous variables were reported as means and standard deviations, or medians with interquartile ranges, depending on normality assessed by the Shapiro–Wilk test, and categorical variables were summarized as frequencies and proportions. Between-group comparisons were performed using the student’s *t* test or the Mann–Whitney *U* test for continuous variables and the chi-square or Fisher’s exact test for categorical variables, as appropriate.

In addition, a negative binomial regression model was applied to assess whether the total duration of mechanical ventilation and the use of high-flow tracheostomy therapy were independently associated with post-tracheostomy mechanical ventilation duration. Model fit was evaluated using the Akaike Information Criterion (AIC) and the dispersion parameter to ensure the absence of overdispersion. All statistical analyses were conducted in R (version 4.4.1) using the RStudio interface (version 2024.09.0–375.0).

## Results

**Fig. 1:**
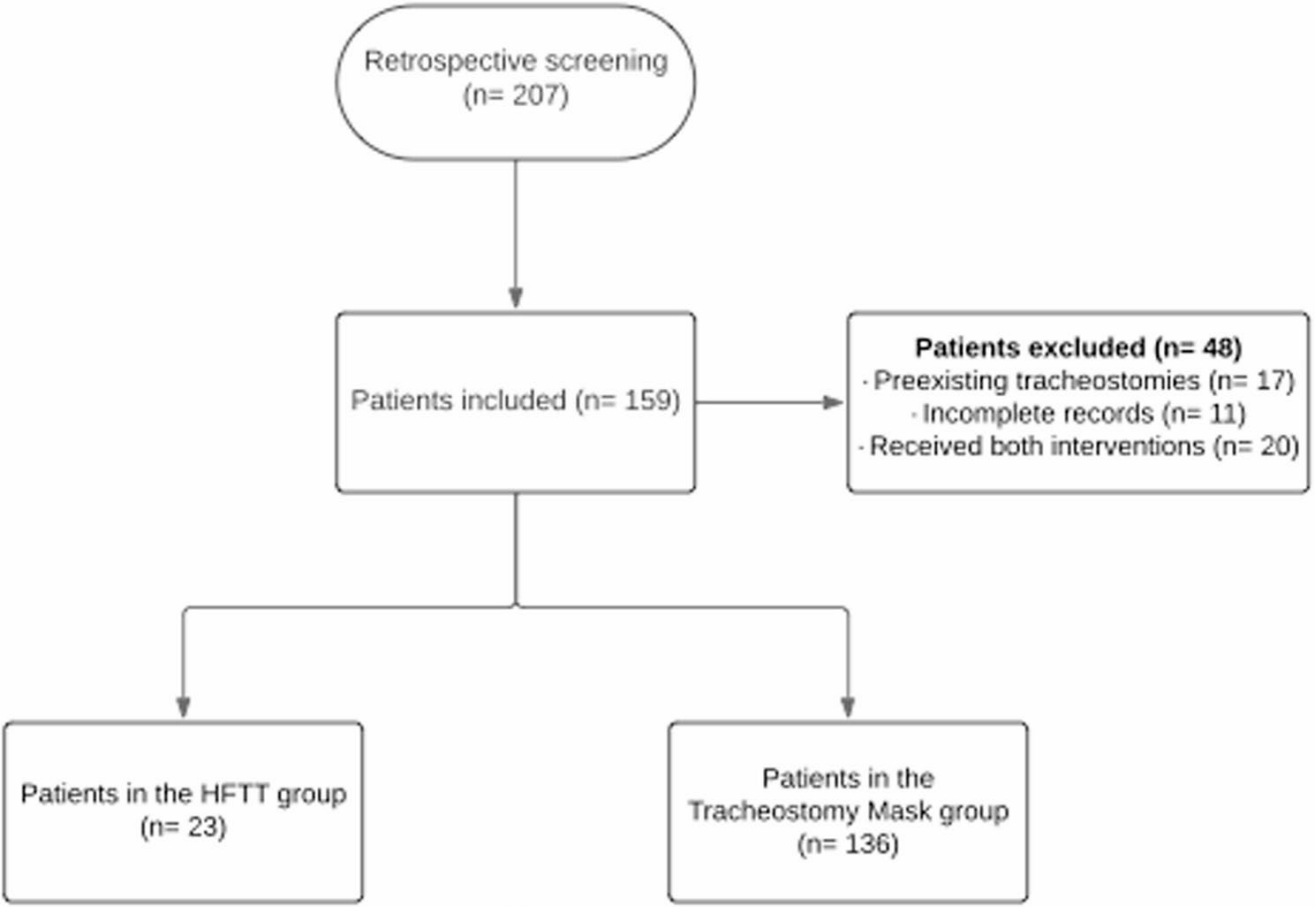
Flowchart diagram

A total of 207 patients were initially screened for eligibility. After applying the inclusion and exclusion criteria, 48 were excluded: 17 due to a preexisting tracheostomy, 11 because of incomplete medical records, and 20 who received both high-flow tracheal therapy (HFTT) and tracheostomy mask interventions (Fig. 1). Following propensity score matching, 65 patients were included in the final analysis, comprising 19 in the HFTT group and 46 in the tracheostomy mask group.

**Fig. 2:**
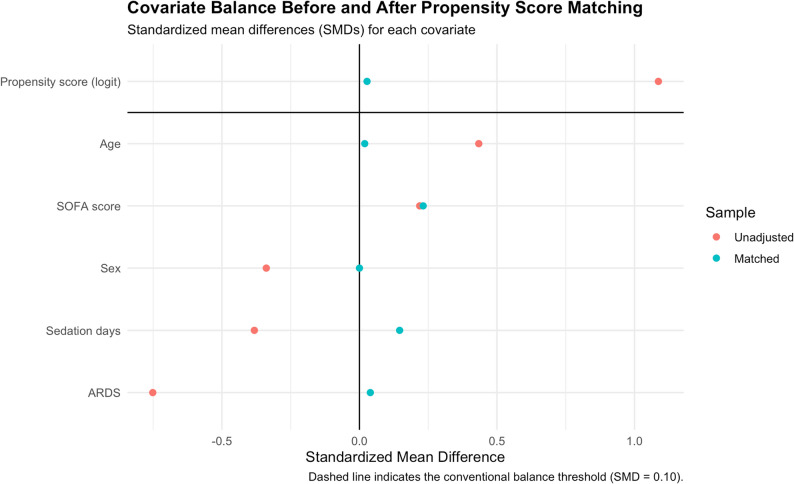
Covariate balance before and after propensity score matching. Each point represents the standardized mean difference (SMD) for an individual covariate before (red) and after (blue) matching. The dashed vertical line at SMD = 0.10 indicates the conventional threshold for acceptable balance

Baseline characteristics were comparable between the HFTT and tracheostomy mask groups, with no statistically significant differences observed (Table 1; Fig. 2).

**Table 1:** Demographic and clinical differences among the tracheostomy mask and high-flow tracheal therapy groups

Variable	HFTT group (*n* = 19)	Tracheostomy mask group (*n* = 46)	*p*-value
Age (years), median (IQR)	67.0 (53.0–72.0)	64.5 (44.2–69.8)	0.46
Sex, n (%)			1.0
Female	6 (31.6%)	16 (34.8%)	
Male	13 (68.4%)	30 (65.2%)	
Diagnosis, n (%)			0.07
Neurological	7 (36.8%)	10 (21.7%)	
Medical	6 (31.6%)	19 (41.3%)	
Polytrauma	1 (5.3%)	12 (26.1%)	
Postoperative	2 (10.6%)	3 (6.5%)	
Cardiovascular	3 (15.8%)	2 (4.3%)	
SOFA score, median (IQR)	4 (2–7)	4 (2–6)	0.34
APACHE II score, mean (SD)	12.7 (5.14)	11.0 (6.27)	0.29
SAPS II score, mean (SD)	36.6 (12.6)	32.4(11.4)	0.20
ARDS, n (%)	4 (21.1%)	11 (23.9%)	1
Vasopressor use, n (%)	18 (94.7%)	41 (89.1%)	0.6
Vasopressor duration (days), median (IQR)	7 (3–10.5)	5.0 (3–10.8)	0.73
Extubation failure, n (%)	5 (26.3%)	6 (13.0%)	0.27
Early tracheostomy, n (%)	4 (21.1%)	20 (43.5%)	0.10
Sedation duration (days), median (IQR)	18 (14–26.5)	18 (13–26)	0.9
Neuromuscular blocker use, n (%)	5 (26.3%)	18 (39.1%)	0.40
Mechanical ventilation duration (days), median (IQR)	28 (22–32.5.5)	21.5 (14.2–27)	0.02
Post-tracheostomy ventilation duration (days), median (IQR)	12 (8.5–19)	11 (7–15)	0.47
Prolonged mechanical ventilation, n (%)	18 (94.7%)	32 (69.6%)	0.05
ICU length of stay (days), median (IQR)	40 (33–49)	33.5 (25–44.8)	0.12
Hospital length of stay (days), median (IQR)	7 (1–15)	8 (1–24)	0.43
In-hospital mortality, n (%)	3 (15.8%)	5 (10.9%)	0.68

Data are presented as median (interquartile range) or mean (standard deviation) for continuous variables, and n (%) for categorical variables. P-values correspond to comparisons between the high-flow tracheal therapy (HFTT) group and the tracheostomy mask group. No statistical test was applied (—) for variables not considered in formal hypothesis testing

*APACHE II* Acute Physiology and Chronic Health Evaluation II, *ARDS* Acute Respiratory Distress Syndrome, *CI* Confidence Interval, *HFTT* High-flow tracheal therapy, *HR* Hazard Ratio, *ICU* Intensive Care Unit, *IQR* Interquartile Range, *SAPS II* Simplified Acute Physiology Score, *SOFA* Sequential Organ Failure Assessment

**Fig. 3:**
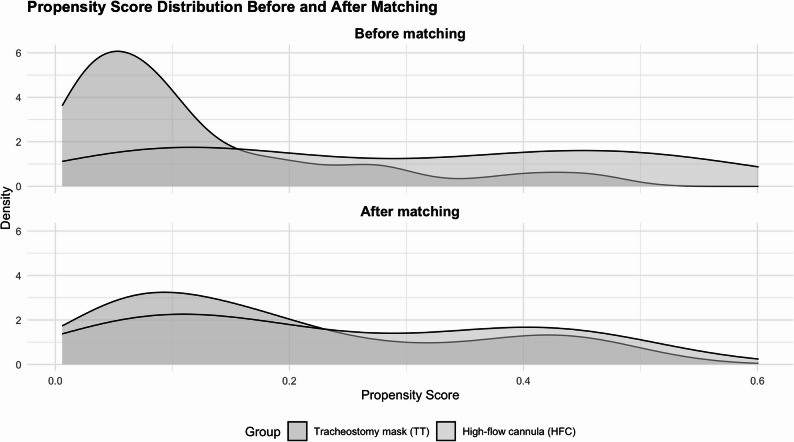
Propensity score distributions before and after matching. Density plots of the estimated propensity scores for both study groups—high-flow tracheal therapy (HFTT) and tracheostomy mask (TT)—before (upper panel) and after (lower panel) propensity score matching

No evidence of multicollinearity was identified among the variables included in the propensity score model, as assessed by the variance inflation factor (VIF) (see Supplemental Digital Content 1, Table S1). The propensity score distribution after matching showed substantial overlap, confirming that the matching procedure effectively improved comparability between treatment groups (Fig. 3). Covariate balance was satisfactory, as indicated by standardized mean differences (SMDs) below the conventional threshold for meaningful imbalance (SMD < 0.1) (Table 2).

**Table 2:** Covariate balance before and after propensity score matching

Variable	Mean (Treated)	Mean (Control)	Std. Mean Diff.	Var. Ratio	eCDF Mean	eCDF Max
Before Matching						
Propensity score (distance)	0.29	0.12	0.90	2.64	0.28	0.46
Age (years)	64.61	56.84	0.42	1.16	0.13	0.31
Sex (1 = male, 2 = female)	1.26	1.42	−0.35	0.82	0.08	0.16
SOFA score	4.87	4.21	0.20	1.36	0.05	0.14
ARDS (0/1)	0.17	0.51	−0.88	—	0.33	0.33
Sedation days	19.13	24.34	−0.41	0.78	0.09	0.30
After Matching						
Propensity score (distance)	0.24	0.23	0.02	1.05	0.01	0.07
Age (years)	61.00	60.66	0.02	1.28	0.05	0.15
Sex (1 = male, 2 = female)	1.32	1.32	0.00	1.03	0.00	0.00
SOFA score	5.11	4.40	0.22	1.33	0.05	0.15
ARDS (0/1)	0.21	0.19	0.05	—	0.02	0.02
Sedation days	21.00	19.01	0.16	1.33	0.05	0.13

Covariate balance between the high-flow tracheostomy therapy (HFTT) and tracheostomy mask groups improved substantially after propensity score matching. All standardized mean differences were below 0.25, and variance ratios were close to 1.0, indicating adequate balance and comparability between groups

*Abbreviations*: *SMD* Standardized mean difference, *VR* Variance ratio, *eCDF* Empirical cumulative distribution function, *SOFA* Sequential Organ Failure Assessment, *ARDS* Acute respiratory distress syndrome, *ESS* Effective sample size

### Differences between the HFTT and tracheostomy masks

All baseline demographic and clinical variables were comparable between groups, with no statistically significant differences observed in disease severity, diagnostic categories, or ICU management parameters. The primary outcome, post-tracheostomy ventilation duration, did not differ significantly between the HFTT and tracheostomy mask groups (*p* = 0.47). In contrast, the total duration of mechanical ventilation was longer among patients treated with high-flow tracheostomy therapy (*p* = 0.02) (Table 1). All other variables, including sedation and vasopressor use, and ICU and hospital lengths of stay, were similar between groups.

Because the only variable differing between groups was the total duration of mechanical ventilation, a negative binomial regression analysis was performed to determine whether this factor—or the use of HFTT—was associated with the duration of weaning after tracheostomy. Neither HFTT use (β = 0.01 ± 0.22, *p* = 0.96) nor total mechanical ventilation duration (β = − 0.005 ± 0.007, *p* = 0.50) were significantly associated with post-tracheostomy ventilation duration. The model demonstrated adequate fit (AIC = 485.8), and the dispersion parameter (θ = 1.84 ± 0.34) indicated no evidence of overdispersion.

## Discussion

In this propensity-matched cohort of tracheostomized patients undergoing prolonged weaning, HFTT did not shorten the duration of mechanical ventilation after tracheostomy compared with conventional oxygen via tracheostomy mask. After matching on key prognostic factors and confirming adequate covariate balance, between-group comparisons showed no significant difference in post-tracheostomy weaning time, and a negative binomial regression model found no independent association between HFTT use and the primary outcome. Although total mechanical ventilation time was longer in patients receiving HFTT, this variable was not related to weaning duration, suggesting possible confounding by indication (e.g., selection of HFTT in patients perceived as more difficult to wean) rather than a causal effect.

Physiologically, HFTT could plausibly facilitate weaning by delivering heated, humidified high flows that reduce dead space and airway resistance, provide a small degree of continuous positive pressure, and improve mucociliary function and comfort [6, 9, 11, 12, 14]. Some crossover studies reported more favorable breathing patterns with HFTT (higher tidal volume, lower respiratory rate, and a lower RR/VT ratio) without an increase in inspiratory effort [6]. However, other physiological investigations have not demonstrated reductions in effort or neuro-ventilatory drive versus T-tube or mask oxygen [9, 15]. The modest, flow-dependent rise in airway pressure achieved with HFTT—typically well below 1 cmH₂O even with substantial increases in flow—may limit clinically meaningful effects on weaning mechanics in tracheostomized patients [7, 8, 16]. Thus, while HFTT can improve oxygenation and gas conditioning [8, 10–12], these benefits may not translate into a consistently shorter post-tracheostomy ventilation duration, as observed here. Importantly, the only randomized trial exploring a decannulation strategy based on high-flow oxygen—by Hernández Martínez et al. [17]—demonstrated a shorter time to decannulation without increased failure rates.

Our results differ in that HFTT did not accelerate weaning in a broader, real-world population, suggesting that such benefits may depend on protocolized approaches and careful patient selection.

Our findings add clinical context to a literature dominated by short-term physiological endpoints. They suggest that routine use of HFTT solely to accelerate post-tracheostomy weaning may be unwarranted. Instead, HFTT might be individualized for indications where its known advantages are most relevant—e.g., secretion burden, patient comfort, and gas humidification, or in scenarios exploring speech-valve compatibility and patient tolerance [10–12, 18]—while acknowledging that a general effect on weaning speed was not demonstrated in this cohort.

This interpretation aligns with large-scale data from Zaga et al. [19], who identified that decannulation outcomes are driven not only by the oxygenation technique but also by patient and system-level factors, such as neurological status, secretion management, and institutional practices.

This study has some limitations. First, the retrospective, single-center design is susceptible to confounding, even after propensity score matching and balance diagnostics. Several clinically relevant variables that influence the weaning process—such as sedation depth, secretion burden, cough strength, diaphragm function, gas exchange indices (FiO₂, PaO₂/FiO₂), pressure support level, and the presence of ICU-acquired weakness—were not consistently available in the electronic health records. Therefore, these data could not be incorporated into the matching process, which may have introduced residual confounding despite the use of propensity adjustment. Second, the heterogeneity of the exposure (flow settings ranging from 20 to 60 L/min, intermittent alternation with ventilator support, and variable cuff status) and the absence of a standardized weaning protocol may have obscured a potential effect. Unlike centers implementing structured decannulation protocols such as those described by Pandian et al. [20], our institution lacked a unified guideline, and modality selection depended largely on clinician judgment and equipment availability.

Third, the sample size—particularly in the HFTT arm—limited the power for subgroup analyses (e.g., ARDS or neurological injury) and for exploring dose–response effects by flow rate. Strengths include a clearly defined primary outcome, comprehensive balance diagnostics, and the use of a propensity-based approach that improves comparability between groups in real-world clinical practice.

Furthermore, multidisciplinary coordination has been shown to significantly improve safety and reduce weaning times in tracheostomized patients [21]. Although such structured collaboration is progressively being adopted in our institution, variability among ICUs may have influenced outcomes and should be considered when interpreting these findings.

Adequately powered, multicenter randomized trials are needed to determine whether protocolized HFTT (with prespecified flows and co-interventions) improves weaning-related outcomes. Embedded mechanistic substudies (e.g., diaphragmatic ultrasound, EAdi, patient-reported comfort) and prespecified subgroups (ARDS, neurological) will help clarify for whom—and under what conditions—HFTT may be beneficial.

## Conclusions

In this propensity-matched cohort, high-flow tracheal therapy did not reduce the duration of mechanical ventilation after tracheostomy compared with conventional oxygen via tracheostomy mask. Until prospective evidence shows otherwise, HFTT use should be individualized for comfort and humidification needs rather than expected to universally accelerate post-tracheostomy weaning.

## Supplementary Information

Supplementary Material 1.

## Data Availability

The datasets generated and analyzed during the current study are available in the institutional repository of the Research Department. Data will be shared upon reasonable request to the corresponding author.

## Abbreviations

ADRS: Acute respiratory distress syndrome
APACHE-II: Acute physiology and chronic health evaluation II
CI: Confidence interval
HFTT: High-flow tracheal therapy
HR: Hazard ratio
ICU: Intensive care unit
IQR: Interquartile range
RR: Respiratory rate
SAPS II: Simplified acute physiology score
SD: Standard deviation
SOFA: Sequential organ failure assessment
STROBE: Strengthening the reporting of observational studies in epidemiology
Tv: Tidal volume
