# Supplementary material for: High-flow tracheal therapy vs. tracheostomy mask in weaning: insights from a propensity-matched cohort
Source: BMC Pulm Med. 2025 Nov 21;26:9. doi: 10.1186/s12890-025-04037-6 (PMC12777010; doi:10.1186/s12890-025-04037-6)
Supplement: Supplementary file 1 — Supplementary Material 1. [file 12890_2025_4037_MOESM1_ESM.docx]

**Table S1. Assessment of Multicollinearity in the Propensity Score Model**

| **Variable** | **VIF** | **Interpretation** |
| --- | --- | --- |
| Age | 1.18 | No evidence of multicollinearity |
| Sex | 1.04 | No evidence of multicollinearity |
| SOFA score | 1.05 | No evidence of multicollinearity |
| ARDS | 1.84 | No evidence of multicollinearity |
| Sedation days | 1.65 | No evidence of multicollinearity |

**Table S1. Assessment of Multicollinearity in the Propensity Score Model.**

**Abbreviations:** VIF = variance inflation factor; SOFA = Sequential Organ Failure Assessment; ARDS = acute respiratory distress syndrome.

**Note:** All VIF values were below 2.0, indicating the absence of significant multicollinearity among covariates included in the propensity score model. This confirms the stability and reliability of the estimated propensity scores.
